# Screening of osteoporosis in patients with ANCA-associated vasculitis (AAV) is quite low: results from a country-wide study

**DOI:** 10.1007/s11739-026-04349-3

**Published:** 2026-04-07

**Authors:** Tuba Demirci Yildirim, Esra Erpek, Bahar Özdemir Ulusoy, Gullu Sandal Uzun, Ayten Yazici, Rıza Can Kardaş, Duygu Sahin, Duygu Sevinc Ozgur, Timucin Kasifoglu, Melih Kiziltepe, Pınar Akyüz Dağlı, Ozge Karakok, Emine Uslu, Arif Babayigit, Elif Durak Ediboglu, Ertugrul Cagri Bolek, Hasan Kocaayan, Gizem Ayan, Bugu Bulat, Busra Firlatan, Berkan Armagan, Gökçe Kenar, Abdulsamet Erden, Hamit Küçük, Ali Sahin, Cemal Bes, Askin Ates, Ayse Cefle, Fatma Alibaz-Oner, Mehmet Engin Tezcan, Haner Direskeneli, Ahmet Omma, Servet Akar, Omer Karadag, Fatos Onen, Turkish Vasculitis Study Group (TRVaS), ANKARA, Turkiye

**Affiliations:** 1 Division of Rheumatology and Immunology, Department of Internal Medicine, Faculty of Medicine, Dokuz Eylul University, Izmir, Turkey; 2 Division of Rheumatology, Department of Internal Medicine, Faculty of Medicine, Izmir Katip Celebi University, Izmir, Turkey; 3 Ankara Bilkent City Hospital, Division of Rheumatology, Department of Internal Medicine, University of Health Sciences, Ankara, Turkey; 4 Division of Rheumatology, Department of Internal Medicine, Vasculitis Research Centre, Faculty of Medicine, Hacettepe University, Ankara, Turkey; 5 Division of Rheumatology, Department of Internal Medicine, Faculty of Medicine, Kocaeli University, Kocaeli, Turkey; 6 Division of Rheumatology, Department of Internal Medicine, Faculty of Medicine, Gazi University, Ankara, Turkey; 7 Division of Rheumatology, Department of Internal Medicine, Kartal Dr. Lütfi Kırdar City Hospital, Istanbul, Turkey; 8 Division of Rheumatology, Department of Internal Medicine, Basaksehir Cam and Sakura City Hospital, University of Health Sciences, Istanbul, Turkey; 9 Division of Rheumatology, Department of Internal Medicine, Faculty of Medicine, Eskisehir Osmangazi University, Eskisehir, Turkey; 10 Division of Rheumatology, Department of Internal Medicine, Faculty of Medicine, Erciyes University, Kayseri, Turkey; 11 Division of Rheumatology, Department of Internal Medicine, Faculty of Medicine, Marmara University, Istanbul, Turkey; 12 Division of Rheumatology, Department of Internal Medicine, Faculty of Medicine, Ankara University, Ankara, Turkey; 13 Division of Rheumatology, Department of Internal Medicine, Faculty of Medicine, Cumhuriyet University, Sivas, Turkey

**Keywords:** ANCA, Associated vasculitis, Osteoporosis, Screening, Bone mineral density, Dual, Energy X, Ray absorptiometry

## Abstract

Patients with ANCA-associated vasculitis (AAV) are at increased risk of osteoporosis due to multiple disease- and treatment-related factors. However, real-world data on osteoporosis screening practices in this population remain limited. The aim of this study was to assess osteoporosis screening practices and the prevalence of reduced bone mineral density (BMD) in patients with AAV using a nationwide registry, and to identify factors associated with osteoporosis. This nationwide, retrospective, web-based study was conducted using the Turkish Vasculitis Study Group (TRVaS) database, including patients diagnosed with AAV between January 2000 and December 2023. Patients were evaluated for osteoporosis screening using dual-energy X-ray absorptiometry (DXA) and categorized according to BMD status. Clinical characteristics and potential risk factors were analyzed, and multivariable logistic regression was performed to identify independent predictors of osteoporosis. A total of 528 patients were included (median age 56 years [interquartile range 43–66]; 57.8% male). Of these, 36.2% underwent DXA screening; among screened patients, 40.8% had osteoporosis and 38.7% had osteopenia. In multivariable analysis, older age (odds ratio [OR] 1.03, *p* = 0.020) and the presence of at least one additional osteoporosis risk factor (OR 2.25, *p* = 0.049) were independently associated with osteoporosis. This nationwide registry-based study highlights a gap between the recognized risk of osteoporosis in patients with AAV and its implementation in real-world clinical practice. These findings underscore the need for improved screening and comprehensive bone health management strategies in this population.

## Introduction

Antineutrophil Cytoplasmic Antibody (ANCA)-associated vasculitis (AAV) affects small and medium-sized vessels and involves the lungs, kidneys, skin, heart, and nervous system. A combination of glucocorticoids and other immunosuppressive agents is the standard of care in new-onset and relapsing AAV [1]. Glucocorticoids are used for longer and at higher doses in AAVs than in other rheumatic diseases because of the risk of fatal course and the fear of relapse [2]. After the remission-induction phase of treatment, AAV evolves into a chronic disease, and management of comorbid conditions associated with the disease may require a better follow-up.

Osteoporosis, one of the most common comorbid conditions in patients with AAV, is associated with decreased quality of life [3, 4]. Patients with AAV have an increased risk of osteoporosis due to not only the use of long-term glucocorticoids but also chronic inflammation [5]. Among the many morbidities in patients with AAV, such as hypertension, diabetes mellitus, and cardiovascular disease, osteoporosis has the highest relative risks difference between AAV patients and the general population [6]. The prevalence of osteoporosis varies considerably across geographic regions. Population-based studies report prevalence rates ranging from 7.3% in men and 38% in women aged ≥ 50 years in Korea to a global prevalence of approximately 18.3% in meta-analyses [7, 8]. In the Eastern Mediterranean region, osteoporosis prevalence has been estimated at 20.5% in men and 24.4% in women [9]. In Turkey, the FRACTURK study reported that the prevalence of osteoporosis at the femoral neck was 7.5% in men and 33.3% in women aged 50 years or older. However, data specifically evaluating osteoporosis prevalence or screening practices in patients with ANCA-associated vasculitis remain limited [10].

Despite the well-recognized increased risk of osteoporosis in patients with AAV, particularly due to chronic inflammation and long-term glucocorticoid therapy, real-world data regarding osteoporosis screening practices and bone mineral density assessment in this population remain limited. Most previous studies have primarily focused on fracture risk or bone density outcomes in selected cohorts, while data evaluating how frequently patients with AAV are screened for osteoporosis in routine clinical practice are scarce. Therefore, the present study aimed to evaluate osteoporosis screening practices and the prevalence of reduced bone mineral density in patients with AAV using data from the Turkish Vasculitis Study Group (TRVaS) registry, a country-wide web-based database established in 2020 [11]. In addition, we sought to identify clinical factors associated with osteoporosis and to examine changes in bone mineral density during follow-up in this population.

## Material and methods

Patients who were ≥ 18 years old and diagnosed as having AAV between January 2000 and December 2023 were included. Patients with a known diagnosis of osteoporosis prior to the diagnosis of AAV were excluded from the study (*n* = 14). Information on the demographic and clinical characteristics of the patients, the treatments they used, and whether they were screened for osteoporosis by the dual-energy *X*-ray absorptiometry (DXA) were obtained using a special page for this project in TRVaS.

Osteoporosis diagnosis was made based on bone mineral density (BMD) through a DXA scan according to World Health Organization (WHO) criteria. Osteoporosis was defined as a BMD of T-score ≤  − 2.5 SD at the lumbar spine, femoral neck, or total hip, and osteopenia as a BMD of T-score between − 1.0 and − 2.5 SD [12].

Risk factors for the development of osteoporosis were defined as having an osteoporotic fracture, a family history of osteoporotic fracture, postmenopausal status in women, alcohol consumption, smoking, malignancy, hyperthyroidism, hypogonadism, anorexia nervosa, chronic renal failure, immobilization, primary biliary cirrhosis, hemochromatosis, gastrectomy, malnutrition, long-term proton pump inhibitor use, and the use of low molecular weight heparin [13]. All patients received long-term glucocorticoid therapy as part of standard AAV treatment (> 3 months at a dose > 5 mg/day prednisolone equivalent). Detailed information regarding the exact duration of glucocorticoid therapy was not systematically recorded in the registry. Therefore, cumulative glucocorticoid dose categories were used to describe glucocorticoid exposure in the study population.

The initiation of antiresorptive therapy was based on physician discretion and local clinical practice in participating centers, generally following national or international osteoporosis management guidelines. In most cases, treatment was initiated in patients diagnosed with osteoporosis by DXA. In selected patients with osteopenia, antiresorptive therapy could also be started if additional fracture risk factors were present.

### Ethical considerations

This study was approved by the Ethics Committee of Hacettepe University (approval number: GO 21/198). Due to the study's retrospective nature, informed consent was not obtained.

### Statistical analysis

Descriptive statistics are presented as medians with interquartile ranges (IQRs) for continuous variables and as frequencies and percentages for categorical variables. Comparisons between groups were performed using the Mann–Whitney *U* test for continuous variables and the chi-square or Fisher's exact test for categorical variables.

Patients were categorized into two groups: those with osteoporosis and those without (including osteopenia and normal BMD). A univariate analysis was performed to identify potential risk factors for osteoporosis. Variables with a *p* value < 0.25 in the univariate analysis and clinically relevant factors (such as age, sex, disease duration, body weight at diagnosis, and the presence of at least one osteoporosis risk factor) were included in a multivariable logistic regression model to assess independent predictors of osteoporosis development. This approach allowed adjustment for potential confounding variables. Since all patients received long-term glucocorticoid therapy, glucocorticoid use was not included as a binary variable in the primary multivariable model. Instead, cumulative glucocorticoid dose was included in the multivariable model to evaluate its potential role as a confounding factor. Odds ratio (OR) with 95% confidence intervals (CI) were reported. A *p* value < 0.05 was considered statistically significant.

Statistical analyses were performed using the Statistical Package for the Social Sciences (SPSS) version 26.0 (IBM Corp., Armonk, NY, USA).

## Results

A total of 528 AAV patients (369 with Granulomatous Polyangiitis, 67 with Microscopic Polyangiitis, 46 Eosinophilic Granulomatous Polyangiitis, and 46 unclassified AAV) were included in this study. The median age was 56 (IQR, 43–66); 305 (57.8%) were male. All patients received long-term glucocorticoid therapy (> 3 months at a dose > 5 mg/day prednisolone equivalent). The distribution of cumulative glucocorticoid doses in the study population is presented in Table 1. The distribution of immunosuppressive agents was 339 (64.2%) cyclophosphamide, 266 (50.4%) azathioprine, 151 (28.6%) rituximab, 95 (18.0%) methotrexate and 78 (14.8%) mycophenolate mofetil. 213 (40.3%) patients had at least one additional risk factor for osteoporosis other than long-term glucocorticoid use (Table 1).

**Table 1:** Clinical characteristics related to osteoporosis in patients with AAV

	All Patients (*n* = 528)
Age, median, years, (IQR)	56 (43–66)
Gender, male, *n* (%)	305 (57.8)
Disease duration, median, months, (IQR)	33 (10–72)
Body mass index (BMI), median, kg/m^2^, (IQR)	26.0 (22.4–29.0)
Having at Least One Risk Factor for Osteoporosis*, *n* (%)	213 (40.3)
Risk Factors for Osteoporosis, *n* (%)	
Menopause	145 (27.5)
Long-term use of PPI	162 (30.7)
Chronic renal failure	70 (13.2)
Low molecular weight heparin	17 (3.2)
Pathological bone fracture	19 (3.6)
Family history of pathological bone fractures	7 (1.3)
Hypothyroidism	12 (2.3)
Alcohol	12 (2.3)
Malignancy	10 (1.9)
Immobilization	5 (0.9)
Hypogonadism	4 (0.8)
Others (one case for each Anorexia nervosa Primary biliary cirrhosis Hemochromatosis Gastrectomy Malnutrition	
Cumulative glucocorticoid dose, *n* (%)	
500–1000 mg	46 (8.7)
1000–2000 mg	69 (13.1)
2000–4000 mg	73 (13.9)
4000–6000 mg	122 (23.1)
6000–8000 mg	71 (13.4)
> 10.000 mg	147 (27.8)

*AAV* Antineutrophil cytoplasmic antibody (ANCA)-associated vasculitis, *BMI* Body mass index, *DXA* Dual-energy X-ray absorptiometry, *IQR* Interquartile range, *PPI* Proton pump inhibitor

*Risk factors other than use of long-term glucocorticoids

During a median disease duration of 33 months (IQR, 10–72 months), DXA was performed in 191 (36.2%) patients. The median time from AAV diagnosis to DXA scan was 21 months (IQR, 4–55 months. Only 78 (14.7%) patients had a DXA scan within the first year after the AAV diagnosis. Patients scanned with DXA had similar demographic and clinical characteristics to unscreened patients, except for AAV duration and having at least one additional risk factor for osteoporosis. The rate of scanning for osteoporosis was found to be higher in patients who had at least one additional risk factor for osteoporosis (57.1% vs. 29.4%, *p* < 0.001) (Table 2).

**Table 2:** Comparison of AAV patients scanned and not scanned for osteoporosis

	Scanned for Osteoporosis (*n* = 191)	Not Scanned for Osteoporosis (*n* = 337)	*P* Value
Age, median, years, (IQR)	60 (40–68)	55 (41–65)	0.099
Gender, male, *n* (%)	106 (55.5)	199 (59.1)	0.427
GPA, *n* (%)	139 (72.8)	219 (67.6)	NA
MPA, *n* (%)	22 (11.5)	43 (13.3)	
EGPA, *n* (%)	23 (12.0)	23 (7.1)	
Unclassified AAV, *n* (%)	7 (3.7)	39 (12.0)	
Disease duration, median, months, (IQR)	44 (20–84)	24 (6–68)	0.005
At least one risk factor for osteoporosis*, *n* (%)	109 (57.1)	104 (30.9)	< 0.001

*AAV* Antineutrophil cytoplasmic antibody (ANCA)-associated vasculitis, *BMI* Body mass index, *EGPA* Eosinophilic granulomatosis with polyangiitis, *GPA* Granulomatosis with polyangiitis, *IQR* Interquartile range, *MPA* Microscopic polyangiitis

*Risk factors other than use of long-term glucocorticoids

Osteoporosis was diagnosed in 78 (40.8%) patients, and osteopenia in 74 (38.7%). Only 39 (20.5%) patients had normal BMD. Patients with osteoporosis were older (median, 64 vs. 54 years, *p* < 0.002) and had lower body weight (median, 68 vs. 74 kg, *p* = 0.03) compared to patients without osteoporosis. Fifty-nine patients (30.9%, 59/191) underwent more than one DXA scan during their follow-up period. The median interval between two DXA measurements was 43 months (IQR: 24–72 months). Among them, BMD decreased in 37 (62.7%), was stable in 19 (32.2%), and improved slightly in only three (5.1%) patients.

Multivariable logistic regression analysis showed that the presence of at least one additional osteoporosis risk factor was independently associated with osteoporosis (OR 2.25, 95% CI 1.09–5.17, *p* = 0.049). The OR for advanced age was found to be 1.03 (95% CI 1.01–1.06, *p* = 0.020). Having a higher body weight at diagnosis has decreased slightly the risk of osteoporosis (OR: 0.97, 95% CI 0.95–0.99, *p* = 0.034). Cumulative glucocorticoid dose was not independently associated with osteoporosis (OR 1.02, 95% CI 0.83–1.26, *p* = 0.842). Gender and disease duration were not found to be risk factors for the development of osteoporosis (Table 3). The median 25-OH vitamin D level was lower in patients with osteoporosis than those without, but there was no statistically significant difference (23 vs. 28 ng/ml, *p* = 0.157). The frequency of osteoporotic fractures in patients with osteoporosis was higher than in patients without osteoporosis, but the difference was statistically insignificant (11.5% vs. 7.1%, *p* = 0.249).

**Table 3:** Multivariate analysis for the development of osteoporosis

	OR	95% CI	*P* Value
Age	1.03	1.01–1.06	0.020
Gender, male	0.87	0.38–1.96	0.734
Disease duration	1.00	0.99–1.01	0.648
Body weight at diagnosis	0.97	0.95–0.99	0.034
At least one risk factor for osteoporosis*	2.25	1.09–5.17	0.049
Cumulative glucocorticoid dose	1.02	0.83–1.26	0.842

*CI* Confidence interval, *OR* Odd ratio

*Risk factors other than use of long-term glucocorticoids

The use of calcium and vitamin D supplementation was high across all groups, with 84.6% of patients with normal BMD, 86.5% with osteopenia, and 92.3% with osteoporosis receiving supplementation. Osteoporosis-specific therapies, including oral bisphosphonates, intravenous (IV) bisphosphonates, and denosumab, were utilized primarily in patients with osteoporosis. Among this group, 46.2% received oral bisphosphonates, 17.9% were treated with IV bisphosphonates, and 7.7% were prescribed denosumab. In contrast, these treatments were less frequently prescribed to patients with osteopenia, with 13.5% receiving oral bisphosphonates and no recorded use of IV bisphosphonates or denosumab. No patients with normal BMD received osteoporosis-specific therapies.

## Discussion

In this nationwide study using the TRVaS registry, we evaluated osteoporosis screening practices and the prevalence of reduced bone mineral density in patients with ANCA-associated vasculitis. The main findings of our study were that only approximately one-third of patients underwent DXA screening during follow-up and that a considerable proportion of screened patients had osteoporosis or osteopenia. These findings highlight a potential gap between guideline recommendations and real-world clinical practice in the management of bone health in patients with AAV.

Patients with AAV are considered at increased risk for osteoporosis due to multiple factors, including chronic inflammation, glucocorticoid exposure, and other disease-related risk factors. Despite this increased risk, osteoporosis remains underdiagnosed in this population. A recent study showed that only 50% of patients aged 40 years and older with AAV had undergone BMD testing. Among those under 40 years, the frequency of screening was found to be as low as 16.6% (3 of 18 patients) [14]. The frequency of osteoporosis screening in other inflammatory diseases where long-term glucocorticoids are used is also quite low. It was reported to be 9.8% in a multicenter study, including patients with chronic respiratory disorders, various rheumatic diseases, and inflammatory bowel disease and 23% in patients who received long-term glucocorticoids due to rheumatoid arthritis [15, 16]. According to current guidelines, the American College of Rheumatology recommends BMD assessment with DXA as soon as possible after initiating long-term glucocorticoid therapy in patients aged 40 years and older [17]. However, in our study, only one-third of patients underwent DXA screening, indicating a substantial gap between guideline recommendations and real-world clinical practice. Low adherence to screening guidelines may partly explain the low rate of osteoporosis screening observed in the rheumatologic population.

The underutilization of DXA screening is particularly concerning, given that 40.8% of the screened patients were diagnosed with osteoporosis and 38.7% with osteopenia in our cohort. Previous studies have also demonstrated the substantial burden of osteoporosis in patients with AAV. Sarica et al. reported that patients with AAV have a significantly increased risk of multimorbidity, with osteoporosis demonstrating one of the highest relative risks compared with the general population [6]. Similarly, Englund and colleagues identified osteoporosis as one of the most frequent comorbid conditions in patients with AAV, with an incidence of 47 cases per 1000 patients per year [18]. These findings suggest that without adequate screening strategies, a considerable number of patients with AAV may remain undiagnosed and therefore vulnerable to fractures and other complications associated with reduced bone mineral density.

Several risk factors for osteoporosis, other than glucocorticoid use, appear to be more prevalent in patients with AAV compared with the general population, and osteopenia was reported to be more common in drug-naive patients with AAV [19]. In our study, 40% of patients had at least one additional osteoporosis risk factor other than glucocorticoid use. The presence of at least one additional risk factor was independently associated with osteoporosis, highlighting the importance of early BMD assessment in these patients. Furthermore, the observation that many patients with osteopenia or normal BMD experienced worsening BMD over time underscores the progressive nature of bone loss and the need for regular monitoring.

Among these factors, body weight emerged as an important predictor in our analysis. In multivariable analysis, lower body weight was associated with an increased risk of osteoporosis. This finding is consistent with previous studies showing that low body weight is a well-established risk factor for reduced BMD [20, 21]. Lower body weight may reflect decreased mechanical loading on bone, which plays an important role in maintaining bone strength and bone remodeling. In addition, lower body weight may also be related to sarcopenia, particularly in patients with chronic inflammatory diseases and advanced age. Sarcopenia, characterized by the loss of skeletal muscle mass and strength, may contribute to bone fragility through reduced mechanical stimulation of bone and impaired muscle–bone interactions. However, information regarding sarcopenia or muscle mass measurements was not systematically recorded in the TRVaS registry; therefore, the potential contribution of sarcopenia to the observed association could not be directly evaluated in this study.

Glucocorticoid exposure is a well-established risk factor for osteoporosis and fractures in patients receiving long-term steroid therapy [22]. However, it did not emerge as an independent predictor in our multivariable model. This may be explained by the relatively homogeneous exposure to long-term glucocorticoid therapy across the cohort, which limits the discriminatory ability of this variable. In addition, the effects of glucocorticoids may overlap with other clinical variables such as age, disease duration, and body weight. Finally, the relatively limited number of patients with available DXA measurements may have reduced the statistical power to detect such an association.

Although calcium and vitamin D supplementation rates were similar across the groups in our study, vitamin D levels remained suboptimal in many patients. This finding suggests that supplementation alone may not be sufficient to ensure adequate vitamin D status in patients with AAV. Long-term glucocorticoid exposure, chronic inflammation, reduced physical activity, and limited sunlight exposure may contribute to insufficient vitamin D levels in this population. According to the 2022 American College of Rheumatology guidelines for the prevention and treatment of glucocorticoid-induced osteoporosis, adequate calcium intake and maintenance of optimal vitamin D levels are essential components of fracture prevention strategies [17]. Therefore, our findings emphasize the importance of not only prescribing supplementation but also regularly monitoring and optimizing vitamin D levels in patients with AAV who are at increased risk for bone loss.

Our study revealed that while osteoporosis-specific therapies, such as bisphosphonates and denosumab, were predominantly utilized in patients with diagnosed osteoporosis, approximately 30% of these patients did not receive any such treatment. This finding raises significant concerns regarding potential gaps in the management of osteoporosis in patients with AAV. Several factors may explain the observed treatment gap. First, physician-related barriers such as underestimation of fracture risk or insufficient familiarity with osteoporosis management guidelines in high-risk populations could play a role. Second, patient-related factors such as complexity of managing comorbid conditions (chronic kidney failure) in AAV and patients concerns about side effects, particularly those associated with bisphosphonates (e.g., gastrointestinal discomfort or rare complications like osteonecrosis of the jaw), may contribute to poor uptake or adherence. In addition, differences in clinical practice patterns across centers may contribute to variability in osteoporosis management. As this was a multicenter registry study, decisions regarding the initiation of osteoporosis-specific therapies were made according to routine clinical practice by the treating physicians at each participating center.

This study has several limitations. First, the lack of a control group from the general population limits the ability to directly compare osteoporosis prevalence between patients with AAV and the general population. Second, we did not evaluate all potential contributors to bone density loss in patients with AAV, such as lifestyle factors (e.g., diet and physical activity) or the potential effects of other medications (e.g., immunosuppressive therapies). Third, the relatively low number of patients with follow-up DXA scans limited our ability to evaluate longitudinal changes in bone mineral density. Finally, the variability in DXA utilization across centers represents an important limitation. Since only a proportion of patients underwent DXA screening, the prevalence of osteoporosis reported in this study reflects the screened population rather than the entire AAV cohort. This may introduce potential selection bias. However, the low rate of DXA screening observed in our cohort suggests that osteoporosis in patients with AAV may remain underdiagnosed in routine clinical practice.

## Conclusion

This nationwide registry-based study highlights an important gap between the recognized risk of osteoporosis in patients with ANCA-associated vasculitis and the real-world implementation of osteoporosis screening strategies. Despite the presence of multiple osteoporosis risk factors, only a limited proportion of patients underwent DXA evaluation. The considerable proportion of reduced bone mineral density among screened patients suggests that osteoporosis may remain underrecognized in routine clinical practice. These findings emphasize the need to improve osteoporosis screening strategies and bone health monitoring in patients with AAV. Prospective studies are needed to better understand the long-term burden of osteoporosis and to define optimal screening and prevention strategies in this population.

## Data Availability

The data supporting this study's findings are available upon reasonable request from the corresponding author.
